# Functional and Economic Role of Some Mediterranean Medicinal Plants in Dairy Ruminants’ Feeding: A Review of the Effects of Garlic, Oregano, and Rosemary

**DOI:** 10.3390/ani15050657

**Published:** 2025-02-24

**Authors:** Piera Iommelli, Anna Antonella Spina, Alessandro Vastolo, Lorenzo Infascelli, Daria Lotito, Nadia Musco, Raffaella Tudisco

**Affiliations:** 1 Department of Veterinary Medicine and Animal Production, University of Naples Federico II, 80137 Napoli, Italy; piera.iommelli@unina.it (P.I.); daria.lotito@unina.it (D.L.); nadia.musco@unina.it (N.M.); raffaella.tudisco@unina.it (R.T.); 2 Department of Health Science, University “Magna Graecia” of Catanzaro, 88100 Catanzaro, Italy; aa.spina@unicz.it; 3 Department of Economics and Law, University of Cassino, 03043 Cassino, Italy; lorenzo.infascelli@unicas.it

**Keywords:** medicinal plants, ruminant nutrition, Mediterranean area, milk production, economic analysis

## Abstract

Botanical and herbal supplements contain diverse compounds that support health and prevent disease by influencing biological pathways. In ruminant nutrition, such supplements are rarely used as most integrations focus on production improvements using synthetic additives. This review explores the potential of Mediterranean herbs, namely, garlic, oregano, and rosemary, in dairy ruminant diets, emphasizing their effects on milk production, antioxidant activity, weaning management, in vitro studies, and economic impacts. Using medicinal plants reduces reliance on synthetic additives, lowers costs, enhances animal health, and boosts productivity. These plants align with sustainable farming practices, enable premium market access, and support local resources to improve performance and reduce livestock’s environmental footprint.

## Simple Summary

Botanical and herbal supplements offer a natural approach to enhancing health and preventing disease by influencing biological pathways. However, their use in ruminant nutrition remains limited as synthetic additives are predominantly employed to improve production. This review investigates the potential of Mediterranean herbs (*Allium sativum* L., *Rosmarinus officinalis* L., and *Origanum vulgare* L.) in dairy ruminant diets, highlighting their effects on milk production, antioxidant activity, and weaning management, as well as insights from in vitro studies and economic implications. These medicinal plants can reduce reliance on synthetic additives, lower costs, and improve both the health and productivity of animals. Furthermore, they align with sustainable farming practices by utilizing local resources, minimizing the environmental footprint of livestock, and enhancing marketability through access to premium markets. Incorporating these herbs in ruminant diets supports eco-friendly and economically viable farming strategies while promoting healthier and more efficient livestock production systems.

## 1. Introduction

The rising global demand for animal protein has led to an increase in ruminant farming, particularly in tropical and developing regions [1]. These farms often rely on natural pastures, supplemented with agro-industrial roughages during dry seasons [2,3]. However, roughages high in lignocelluloses lack sufficient fermentable carbohydrates and nitrogen, leading to poor digestibility, reduced feed intake, and increased methane emissions, which contribute to environmental degradation and energy loss [4,5,6,7]. Improving rumen fermentation is identified as a pivotal strategy to manipulate the microbial flora of the rumen, leading to increased productivity. In livestock production, antibiotics have traditionally been used to prevent disease, address metabolic disorders, and enhance feed efficiency. Due to growing public concerns and bans on antibiotic use in animal feed as growth promoters in the European Union (Directive 1831/2003/EEC, European Commission 2003), alternative strategies are being explored. These include optimizing feeding strategies and breed selection and using synthetic and natural compounds. In their editorial, Ungerfeld and Newbold [8] reiterate that for more productive and sustainable ruminant production, the manipulation of rumen microbial activity is unavoidable. Plant extracts with antimicrobial secondary metabolites, such as saponins and tannins, represent an alternative approach to addressing microbial challenges within the agricultural context [9]. It has been demonstrated that these compounds can regulate rumen fermentation to enhance the utilization of nutrients in ruminants [10]. Several researchers [11,12,13,14,15] have reported that phytochemical-rich plants and their extracts represent promising alternatives to antibiotics and chemical additives for modulating the rumen microflora. Therefore, to enhance production efficiency, optimize animal health, and address sustainability concerns, researchers and farmers have turned to nutraceuticals, i.e., bioactive compounds derived from natural sources with potential health benefits [16]. They represent an alternative feed additive, acting on the immune system as antioxidants, anti-inflammatories, and antimicrobials, thus improving animal health and, consequently, animal productivity. The phytoactive compounds revealed potential support to the immune system, reducing the risk of the development of animal diseases [17]. In particular, their antioxidant activity regulates oxidative stress, which has severe consequences on the immune system functions, leading to an increased incidence of diseases [18]. There is therefore a need to elucidate the various active constituents of herbs and the possible explanation of their mechanism of action so that they can be incorporated into a specific feed formulation with high efficacy and safety after the standardization of the correct dosage regime. Increasing the knowledge base of these plants and their benefits on farms is an opportunity to use local resources to improve animal performance and reduce the environmental impact of livestock production while limiting the use of synthetic drugs in livestock production.

### 1.1. Medicinal Plants

It is evident that plants have been used as drugs in various medical traditions throughout history [19]. Ethnobotanical studies serve as a testament to the extensive use of numerous plant species and the remarkable potential of these substances, as evidenced by their long-standing and widespread use. A wealth of evidence from human medicine and nutrition research demonstrates the preventive and therapeutic efficacy of plants. This evidence also supports the use of plants as supplements on a daily basis to maintain good health [20]. On the other hand, there is a paucity of research in the field of zootechnics that specifically addresses the objective of enhancing production through commercial integration. Nevertheless, they have recently been gaining attention as potential candidates to control parasites in ruminants as their consumption may be linked to good performance and anthelmintic impact. Medicinal plants are defined as plants that have therapeutic properties or beneficial effects on the human or animal body [21] and have already shown their efficacy in ruminants for treating parasitic diseases [22], mastitis [23], and other pathologies [24] or for improving the efficiency of the immune system [25]. Plant natural products comprise a diverse array of bioactive molecules such as flavonoids, tannins, glucosides, alkaloids, saponins, terpenoids, amines, non-protein amino acids, and organosulfur compounds [26]. Since the majority of vegetal species have anti-inflammatory properties, they are usually used to treat a great variety of diseases characterized by inflammation, like infection, trauma, or infestation. In-depth research on the composition and the effects of these plants through both in vitro and in vivo trials has shown how and why they function in a specific manner. In general, most herbs have anti-inflammatory and antioxidant activity, more or less evident in different species, making them suitable to be used as a natural remedy. Nevertheless, several plants have been employed to treat specific diseases, or when used for multiple purposes, they have been prepared in different ways. The versatility of these herbs is due to the multitude of compounds exerting beneficial properties, which provide a single plant with several therapeutic properties. Consequently, the optimal use of vegetal remedies necessitates the utilization of specific pharmaceutical forms to achieve the desired preventive or therapeutic outcomes. By elucidating the composition of herbs and the physicochemical characteristics of their bioactive compounds, it is possible to select the most efficacious method of extraction and administration to elicit a specific effect. A common practice is to use a mix of herbs or their extracted compounds to obtain a synergic effect, meaning that the mixture could enhance the beneficial results of the single herb and sometimes help to correct the detrimental side effects of a specific drug. Vegetal drugs could be administered as a simple powder (loose powder or pills) or used as extractions obtained in different ways (water extract, infusions, dry extraction, and fluid extraction) [20]. In addition to these, there are other less common types of presentation of drugs, like mother tincture, gemmotherapy extracts, and essential oils. The selection of the specific form of administration of a drug represents a pivotal stage in the process of obtaining a specific effect from a given treatment or supplementation. It is essential that this stage is targeted towards a specific goal as different formulations of a drug may result in disparate pharmacological outcomes [27].

### 1.2. Mediterranean Plants

The Mediterranean area is considered to have a great variety of plants, accounting for over 25,000 vegetal species [28]. Lots of Mediterranean countries have traditionally been using some vegetal species (plants, shrubs, or trees) as natural remedies in folk medicine. González-Tejero and his team [29] conducted a survey on the use of plant-based medicine in different Mediterranean countries and were able to take a census of over 950 species, of which more than 400 were identified as medicinal plants. Although these numbers are striking, it is hard to ascertain the real extent of this phenomenon. In fact, in the relatively small area of Cilento, located in the Campania region of Italy, nearly 2000 plants exhibiting vascular activity have been identified [30].

## 2. Research Method of the Review 

This review aims to summarize the knowledge of Mediterranean medicinal plants’ use in ruminants’ feeding. The literature search was performed using Google Scholar, PubMed, and ScienceDirect using the following keywords: “ruminants”, “mediterranean plants”, “supplementation”, “integration”, and “feeding” and five different topics of interest (“milk quality”, “antioxidant activity”, “weaning”, “in vitro studies”, and “economic”), selecting only papers published since 2000. Since the basal diet of ruminants already consists of plants that are considered medicinal (i.e., *Medicago sativa* [31] and *Trifolium pratense* [32]), the keywords “supplementation” and “integration” allowed us to focus only on plants used for their highly beneficial properties and not for their nutritional value. Following an initial investigation, only three plants were selected for this review as they were the most extensively researched in the chosen fields. The decision to include in vitro studies was motivated by the increased interest surrounding these experimental models, which facilitate the assessment of dietary regimes within a comparatively reduced timeframe. The in vitro studies are discussed separately as most of the works selected tested a combination of these plants mainly as a blend of essential oils. In the same way, the economic considerations of the use of medicinal plants are discussed, giving an overview of the economic issues related to medicinal plant supplementation. Therefore, below, 5 paragraphs are presented in the Discussion section concerning the effects of garlic (*Allium sativum* L.), rosemary (*Rosmarinus officinalis* L.), and oregano (*Origanum vulgare* L.) supplementation, followed by “in vitro studies” and “economic implications”.

## 3. Garlic

Garlic (*Allium sativum* L.) is a common spice with a multitude of health benefits, primarily due to its diverse array of bioactive compounds, including organic sulfides, saponins, phenolic compounds, and polysaccharides [33]. It is a bulbous plant belonging to the Alliaceae family and it mainly grows in temperate climates. Garlic contains at least thirty-three sulfur compounds, [34] with allicin (allyl 2-propenethiosulfinate or diallyl thiosulfinate) being the principal bioactive one [35]. Specifically, in the work of Bayan et al. (2014) [35], the mechanism of action of allicin has been described thoroughly. It has been established that allicin possesses sulfhydryl-modifying activity, with the capacity to impede the function of sulfhydryl enzymes. Cysteine and glutathione have been observed to counteract the thiolation activity of allicin. Furthermore, experimental evidence has demonstrated the bacteriostatic effects of garlic extract and allicin on certain vancomycin-resistant enterococci [35]. This plant has been widely recognized as a traditional remedy for many disorders and has been used for medicinal purposes for thousands of years [34].

### 3.1. Milk Characteristics

In recent years, a considerable body of research has demonstrated the remarkable biological effects of garlic in animals, including ruminant species. However, few studies have been performed on the potential effects of garlic on milk production and composition. In a study conducted by Yang et al. [36], the effects of garlic essential oils (in a ratio of 5 g/cow per day) on lactating cows were evaluated. The results indicated a tendency towards increased milk fat content (*p* < 0.10). The precise mechanism by which garlic increases milk fat content remains unclear as dry matter intake and fiber digestibility in the rumen were found to be similar between cows supplemented with garlic and those fed the control diet. In contrast, Rossi et al. [37] reported that garlic supplementation (4.35 or 17.39 g/kg of dry matter (DM) of garlic cloves) had no effect on the production and composition of milk in dairy cows. Zakeri et al. [38] reported that the addition of pure garlic bulbs in the diet in a range of 30 to 70 g/kg (DM) for 14 days can effectively reduce the fat concentration in goat milk, probably due to an action of garlic on the production of acetate in the rumen. In fact, some authors [39,40] reported an inhibitory action on acetate production with the addition of garlic-based products. The addition of garlic oil (2 mL/day per goat) to the diet of lactating goats for a period of three months has been demonstrated to be effective [39]. Furthermore, the supplementation of garlic at a dose of 20 g/kg (DM) for approximately five months in the diet of dairy sheep [41] led to an increase in the production of conjugated linoleic acids (CLAs) and omega-3 fatty acids (FAs) in milk, which are beneficial to human health [42,43]. Similarly, Yang and He [44] reported that supplementing dairy cows with garlic oil at a dose of 5 g/day per cow for three weeks can increase the concentration of cis-9, trans-11 CLA in milk. This FA has been shown to have anti-inflammatory, anti-diabetic, and anti-tumor properties [45,46]. In conclusion, it appears that the studies examined utilize disparate garlic-based formulations. It would therefore be beneficial to standardize these in order to identify a more efficacious dose and formulation, with the aim of improving the quality of ruminant milk.

### 3.2. Antioxidant Properties

Garlic (*Allium sativum*) has been demonstrated to possess a number of beneficial properties, including an improvement in nutrient digestibility and antimicrobial, anti-inflammatory, antioxidant, and immunostimulant action in animal nutrition [33]. Garlic and its derived products are rich in bioactive organosulfur compounds such as disulfide, allyl, alliin, allicin, allixin, allyl cysteine, and allylsulfides, which have different patterns of action against free radical damage [47]. Studies have also demonstrated that allyl polysulfides display antioxidant effects in humans and animals [48]. The powder, skin, and leaf of garlic may influence the activity of serum antioxidant enzymes, potentially enhancing the antioxidant status of ruminants [49]. Lee et al. [50] proposed that the inclusion of a combined mixture comprising garlic in the diet of dairy cows may enhance milk yield and health status under mild to moderate heat stress. This suggests that garlic may be a valuable addition to the diet of dairy cows in warm conditions, potentially serving as an alternative anti-stressor. Nevertheless, the impact of various garlic-derived products on antioxidant parameters varies considerably across different species of animals. Consequently, further studies are required to fully elucidate the effective utilization of nutrients [51,52,53].

### 3.3. Weaning

Weaning ruminants is a crucial stage that significantly affects the productivity of adult animals on dairy farms [54,55,56,57]. Kekana et al. [58] showed that feeding 5 g/day of garlic powder to Holstein calves (4 to 42 days old) resulted in higher serum IgG levels compared to the control group (28.0 g/L vs. 23.5 g/L). Neither growth performance nor fecal scores changed. On the other hand, when administered with probiotics, garlic also improved the fecal score and reduced the number of diarrhea episodes. In weaning calves, garlic powder (GP) appears to be beneficial even when administered at lower doses. In fact, Özkaya et al. [59] observed a significant decrease in the oxidative stress index with the administration of 10 mg GP/kg body weight (BW), along with a significant reduction in the incidence of diarrhea and respiratory disease with a dose of 30 mg GP/kg BW in 30 Holstein calves (from 4 days of age to 56 days of age). The fecal score also improved in the study by Ghosh et al. [60], when 36 Holstein cross calves (aged 5 to 60 days) were fed garlic extract supplementation at 250 mg/kg body weight per day. The presence of GP also resulted in a significant increase in mean body weight and feed intake (FI) compared to the control diet. Similar results were observed in weaning lambs and kids. Toghdory et al. [61] used milk enriched with different doses of garlic powder (1.5 or 3 g of garlic powder per day for 45 days) in 24 newborn lambs. The results obtained from this research showed that by adding garlic powder, regardless of the dose, the final weight and feed consumption increased significantly. Additionally, these treatments improved nutrient digestibility and stool consistency. In terms of blood parameters, garlic powder caused a significant decrease in plasma triglycerides, cholesterol, and low-density lipoproteins, while glucose and high-density lipoprotein concentrations were increased. Shokrollahi et al. [62] administered milk fortified with 0, 62.5, 125, and 250 mg of aqueous garlic extract per kg of live body weight per day for 42 days to male goat kids (5–10 days of age). Average daily gain (ADG), BW, red blood cells, lymphocytes, and white blood cells were significantly higher for goats fed the highest dose of garlic compared to the control group. Overall, garlic products, being rich in organosulfur compounds, perform some biological activities that appear to improve the growth performance and diarrheal phenomena of ruminants during weaning. The main dosage and effects on milk production performance and weaning efficiency derived from garlic administration are listed in Table 1.

## 4. Oregano

Oregano (*Origanum vulgare* L.) is an aromatic perennial herb that grows in the Mediterranean and western Eurasia regions. It belongs to the family of Lamiaceae and is mainly used as a spice. Its use as a natural remedy dates back to ancient times as it was used by the Greeks and Romans as an antiseptic and for the treatment of asthma, diarrhea, and other illnesses [64]. Oregano is rich in essential oils (EOs—volatile secondary metabolites of plants) characterizing its odor and flavor, such as carvacrol, thymol, γ-terpinene, and *p*-cymene [63,64], also protecting the plant itself from microorganisms, herbivores, and UVB radiation [65]. The main bioactive compounds present in oregano are carvacrol and its isomer, thymol [66,67]. Carvacrol, due to its hydrophobic nature, has been shown to penetrate bacterial cell membranes, resulting in alterations to membrane permeability. This process has been observed to lead to the leakage of vital ions, including potassium (K^+^) and protons (H^+^), which disrupt essential cellular processes [68]. In a similar manner, thymol acts as an antimicrobial agent by causing perturbations in the lipid fraction of the bacterial plasma membrane. These perturbations are characterized by the induction of the permeabilization and depolarization of the cytoplasmic membrane, which consequently leads to the leakage of intracellular materials [69].

### 4.1. Milk Characteristics

Two varieties of oregano (spp. *vulgare* vs. *hirtum*) were tested on lactating Danish Holstein dairy cows, and the effects on milk fatty acid composition were evaluated [70]. Multiple dosages of the supplementation (18, 36, and 53 or 7, 14, and 21 g of oregano DM/kg of dietary DM, respectively, of spp. *vulgare* vs. *hirtum*) did not result in any changes in the composition of milk or on its acid profile. In addition, Hristov et al. [71] tested much higher dosages (from 250 to 750 g/day) of oregano leaves in the diet of lactating Holstein cows and did not find differences in milk production, composition, and fatty acid profile. However, in vivo studies testing the use of oregano extract as a food supplement in animal nutrition are still scarce [72]. Tekippe et al. [73] found a slight increase in milk fat, although the data were not significant, contrary to what was observed by Santos et al. [74], who found a significant (*p* < 0.05) increase in fat content in milk produced by supplemented dairy cows. In contrast, Kolling et al. [75] found a decrease in milk fat percentage and the fat/protein ratio when oregano (at a dose of 0.056% extract/DM of the diet) was administered to dairy cows. This result is probably attributable to oregano antimicrobial properties that could be responsible for changes in the ruminal flora and, consequently, for variations in the composition and acid profile of the milk. To confirm this hypothesis, these authors found a reduction, although not significant, in some short-chain saturated fatty acids. Works testing oregano in small ruminants are fewer than those on dairy cows. In the work of Paraskevakis [76], 30 g/head/day of ground oregano plants were supplemented to dairy goats’ diet in mid-lactation for 4 weeks, with the aim of evaluating the effects on milk yield. The author found no differences between the control and treated groups and attributed this result to the low daily dosage, a result consistent with previous work on ewes [77], where a similar dosage failed to improve small ruminant performance. The high variability in the compounds described in this discussion and in the dosages has led to conflicting in vivo results, although most studies suggest that oregano is not capable of exerting relevant effects on the feeding of dairy ruminants.

### 4.2. Antioxidant Activity

Oregano is distinguished by the presence of phenolic compounds, including flavonoids and phenolic acids, which are primarily responsible for its antioxidant activity [78,79,80,81,82,83]. The two most important phenolic compounds, corresponding to 78–85% of the oil composition, are carvacrol (2-methyl-5-isopropyiphenol), an aromatic oxygenated monoterpene, and thymol (2-isopropyl-5-methylphenol). These biocomponents have the capacity to neutralize the cascade of free radical reactions [84]. A number of studies have demonstrated that aromatic plants can enhance the efficacy of the antioxidant system by boosting the activity of antioxidant enzymes in rats [85], broiler chickens [86], and fish [87]. Furthermore, some in vivo studies have indicated that the intake of aromatic herbs can positively impact the performance of dairy cows [73,88]. The supplementation of oregano minimizes oxidative damage during the lactation period [76]. The transition period of dairy cows, from late pregnancy to early lactation, is one of the most vulnerable periods of their lives as it increases the incidence of mastitis and reduces milk quality [89]. To reduce these risks, dietary supplementation could be useful, as studied by Stivanin et al. [90], who showed that carvacrol and thymol, when integrated as a feed supplement in dairy cows, have the ability to affect the properties of the cytoplasmic membrane and are able to inhibit bacterial adhesion to mammary epithelial cells, resulting in a reduction in the risk of postpartum mastitis and a decrease in somatic cells in milk.

### 4.3. Weaning

As for calves, essential oils can have a beneficial effect during the weaning period since when they are added to their milk or diet in general, they help to improve the immune system, which is often linked to feed intake, performance, body development, and blood metabolites [91]. In the study by Ansari et al. [79], 57 female Holstein calves (1 d of age; 34.1 ± 0.97 kg BW) received liquid feed (colostrum and milk) without added herbage (CON) or with oregano leaf powder (in ratio of 30 g/day) during the first month of age. Calves receiving oregano had a higher average daily gain, body weight gain, and feed efficiency than animals receiving only milk. Oregano leaf powder supplementation also significantly reduced the duration of diarrhea, and as for blood metabolites, only the blood concentration of triglycerides was higher in the experimental group than in the control group. Triglycerides, along with cholesterol, represent important indicators of lipid metabolism, and their concentrations are correlated with health and early mortality in calves [92]. Different results were obtained by Heisler et al. [93], who evaluated the effects of *Origanum vulgare* extracts (intake of 60 mg/kg BW/day) on the behavior, weight gain, and health indicators of 21 Jersey calves from birth to 60 days of age. Although the presence of oregano did not modify body weight gain, the incidence of diarrhea, health conditions, and other behavioral variables, the authors made an interesting observation regarding rumination: the oregano extract anticipated the occurrence of straw ingestion and, therefore, the first rumination in the treated groups compared to the control group (12 days vs. 19 days). The early onset of rumination is important as it indicates a more developed rumen, capable of absorbing and metabolizing fermentation products, and may lead to early weaning [94]. Encouraging results were also obtained with the administration of oregano EO on neonatal diarrhea syndrome in calves aged less than 15 days [95]. Ninety newborns from dairy farms were assigned to two groups: 45 calves to the group treated with 5% oregano EO (*Origanum vulgare* ssp. *hirtum*) at a dose of 12.5 mg/kg BW once a day up to the age of 10 days and 45 calves as the control group. The incidence of diarrhea was monitored on a daily basis in all animals until they reached the age of 15 days, with a record kept of their fecal score. A significant reduction in mean fecal score, incidence of diarrhea, duration of diarrhea episodes, and severity of diarrhea episodes was observed in the treated group in comparison to the control group during the experimental period. The prevention of diarrhea during weaning, especially in the first 10 days of life, is essential since the majority of diarrhea cases occur in this period. The potential therapeutic effect of oregano essential oil on neonatal diarrhea syndrome may be attributed to its ability to inhibit the overgrowth of coliform bacteria in the small intestine of diarrheal calves. This is due to the essential oil’s strong antibacterial activity against *E. coli* [95,96]. The main dosage and effects on milk production performance and weaning efficiency derived from oregano administration are listed in Table 2.

## 5. Rosemary

Rosemary (*Rosmarinus officinalis* L.) is an evergreen perennial shrub that belongs to the Lamiaceae family and is widely used as a spice, especially in Mediterranean cuisine. Its biological activity is mainly related to its phenolic and volatile compounds, such as carnosol, carnosic acid, and rosmarinic acid, which are particularly abundant in rosemary extract, as well as α-pinene, bornyl acetate, camphor, and eucalyptol, which are present in the essential oil [97]. Carnosic acid and carnosol have been demonstrated to interact with microbial cell membranes, resulting in the disruption of the lipid bilayer. This disruption leads to an increase in membrane permeability, causing the leakage of cellular contents (ions, nucleotides, and proteins) and cell lysis. Furthermore, these compounds have been shown to reduce biofilm biomass by altering the expression of key genes responsible for biofilm development [98]. In folk medicine, it was mainly used for its anti-inflammatory and antimicrobial properties [99].

### 5.1. Milk Characteristics

In Damascus goats, the effect of administering 10 g/head/day of dried rosemary in the diet was investigated to evaluate the potential effects on production performance [100]. Supplementing the diet with rosemary increased (*p* < 0.05) milk yield and milk fat yield (*p* < 0.001). The milk fatty acid profile was also influenced by the supplementation, with an increase in unsaturated fatty acids (UFAs) (*p* < 0.01), monounsaturated fatty acids (MUFAs) (*p* < 0.01), and CLA (*p* < 0.05) and a decrease in saturated fatty acid (SFA) concentration (*p* < 0.01). These effects are postulated to be beneficial in terms of the prevention of coronary artery disease in those who consume dairy products. Additionally, increasing the UFA content of milk is thought to be beneficial, given that these FAs are involved in regulating numerous cellular processes and are associated with the development and function of the immune system [101]. The supplementation of goats with lemongrass and rosemary resulted in an increase in milk yield without a concomitant increase in feed intake. The improved digestibility of nutrients and rumen fermentation observed in these animals may be the main reasons for this increase in milk production. This result may be due to the increase in lactose concentration in milk, which leads to a higher milk yield [102]. Chiofalo et al. [103] observed that administering rosemary extract (in dosages of 600 and 1200 mg/head/day) to dairy sheep diets increased milk production and the concentration of fat, protein, casein, and lactose, as also confirmed in a subsequent study by Chiofalo et al. [104], who tested the inclusion of 1200 mg of rosemary extract in the diet of sheep from the Belice Valley, which led to an increase in the milk protein and fat content. These results are probably due to the presence of phenolic compounds in rosemary, which may be able to improve rumen fermentation processes by creating a strictly anaerobic environment [105]. A more recent study reported less dramatic results in Holstein cow [106] supplemented with 28 g/d of rosemary extract. The authors reported an increase in milk yield, along with a significant improvement in milk protein content (*p* < 0.05). The results demonstrated the potential of dietary supplementation with rosemary in dairy ruminant diets, rendering it a suitable candidate for inclusion as a functional ingredient.

### 5.2. Antioxidant Activity

Rosemary is one of the plants with the highest antioxidant activity [107]. Its use as an antioxidant began in the 1950s, and it has been used in the food industry throughout the world, showing the best protective capacity in animal products [108]. In accordance with Georgantelis et al. [109], Hashemzadeh-Cigari et al. [110] demonstrated that the antioxidant activity of rosemary extract is associated with the presence of several phenolic diterpenes, including carnosic acid, carnosol, rosmarinic acid, rosmanol, and rosmaridiphenol. These compounds break free radical chain reactions by electron donation and metal ion chelation, contributing to the extract’s potent antioxidant activity. Carnosic acid, a major phenolic constituent of and the most abundant compound in rosemary leaves, exhibits potent antioxidant activity, stronger than some synthetic antioxidants [111]. It exhibits a high reactivity towards reactive oxygen species (ROS) and acts as a scavenger, removing ROS [112]. In a study conducted by Jordán et al. [113], it was demonstrated that dietary supplementation with carnosic acid and its derivative carnosol exhibited antioxidant efficacy following ruminal fermentation in lambs. In a study conducted by Michelotti et al. [114], carnosic was directly infused into the veins of transition dairy cows, with a positive effect on inflammation and oxidative stress biomarkers [114].

### 5.3. Weaning

Rosemary, in the form of essential oil, has been tested as a feed additive by Biyik et al. [115], who allocated 40 Holstein calves to four groups, each comprising 10 calves: three groups received three different levels of rosemary essential oil (REO), i.e., 500 mg/day, 1000 mg/day, and 2000 mg/day, while the fourth group was used as control. Calves fed the first two levels of rosemary essential oil supplementation had higher starter intake and ADG. Furthermore, at the beginning of the trial, calves fed the two highest rosemary essential oil levels had the highest serum IgG concentration compared to calves fed the control diet. Different doses of rosemary essential oil were also tested in lambs during the weaning period [116]. In particular, 32 male Merino lambs (78 ± 5 days of age) received the following doses of REO: 0 mg/day (control group), 250 mg/day, 500 mg/day, and 750 mg/day. Unlike the results obtained with dairy calves, no dose of REO influenced the performance of the lambs (live weight gain, ADG, daily feed intake, daily dry matter ingested, and feed conversion ratio). However, the addition of 750 and 500 mg/day of REO to the lamb diet caused a significant increase in the plasma levels of superoxide dismutase (SOD) [116]. To examine the effects of this plant on newborn goat kids, one study [117] tested the effects of including different levels of rosemary extract on 34 goat kids (aged 7 ± 3 days). The trial included one control group and three experimental groups that received milk supplemented with 100, 200, and 400 mg of aqueous rosemary extract per kg of live BW per day for 42 days. No significant differences were observed in the initial BW and ADG, but there was an increase in white blood cells (WBCs) and the amount of globulin (only for the group with the highest level of rosemary extract). These results demonstrate how REO positively influences some serum proteins, including IgG. This increase may be due to the stimulation of B lymphocytes [118]. However, in other studies [119], where they used the active ingredients of rosemary, the positive effects on IgG and IgA concentrations in calves did not occur. The main dosage and effects on milk production performance and weaning efficiency derived from rosemary administration are listed in Table 3.

## 6. In Vitro Studies

The diversity of plants and the challenge of determining appropriate dosages and methods of administration highlight the necessity to increase research on the inclusion of medicinal plants in ruminant diets. In this respect, in vitro studies allow a wealth of information to be obtained and different dosages and pharmaceutical forms to be explored in less time. In vitro methods are advantageous in terms of speed and cost compared to in vivo techniques. Indeed, in vitro protocol advantages involve the ability to run a lot of experimental units at the same time to measure the fermentation kinetics of a single feed and to make comparisons between different feeds or diets. The in vitro cumulative gas production techniques established the ability of microorganisms to ferment carbohydrates in anaerobic conditions [120,121]. The final output analysis included fermentation kinetics, organic matter degradability, and fermentation end-products (e.g., volatile fatty acids and ammonia) [122]. In the last decade, in vitro methods have also been suggested for the analysis of methane production [123]. The in vitro protocols may provide valid preliminary nutritional strategies for reducing greenhouse gas (GHG) emissions in animal production and for evaluating feedstuff, bioactive molecules, or diets that are able to limit methanogenic bacteria. Furthermore, the analysis of chemical composition and in vitro parameters is a complete method of screening feedstuffs and diets before their use, especially when they are new or not well-studied. In addition, the EO and its bioactive compounds have been confirmed to modify ruminal fermentation, with an improvement in energy utilization and a reduction in methane emissions [124,125,126]. They have also been studied as potential converters of ruminal biohydrogenation of dietary lipids, with an improvement in milk and meat characteristics [127,128].

### Garlic, Oregano, and Rosemary

Cavalheiro Junior et al. [129] conducted a study investigating the ruminal degradation kinetics of carbohydrates in lamb diets with varying roughage-to-concentrate ratios and different dosages of garlic and rosemary EOs. The objective was to determine the most suitable dosage of these EOs for optimal carbohydrate degradation. The authors tested three roughage: concentrate ratios (50:50, 40:60, and 20:80) and six dosages of garlic and rosemary EOs (0.0, 0.10, 0.25, 1.0, 1.50, and 2.0 g L^−1^). The results suggested that the inclusion of rosemary and garlic essential oils in the diet of feedlot lambs affected carbohydrate degradation kinetics. Oil dosages did not depend on the roughage: concentrate ratios. Therefore, a ruminal 1.0 g L^−1^ is recommended for both rosemary and garlic essential oils. Cobellis et al. [130] observed that oregano and rosemary EOs mitigated rumen fermentations, reducing methane and ammonia production. The authors studied the effects of increasing the concentrations of oregano (*Origanum vulgare* L.) and rosemary (*Rosmarinus officinalis* L.) EOs on ruminal gas emissions, incubating at 39 °C for 24 h in each bottle with 200 mg substrate (alfalfa hay/corn meal ratio of 1:1) and a 20 mL solution composed of a buffered medium and bovine rumen fluid (1:2). Oregano and rosemary EOs were used at doses of 0 (control dose), 0.5, 1.0, and 1.5 g/L. The results of the study demonstrated that oregano EO, at the greatest level of 1.5 g/L, is a potent inhibitor of ruminal CH_4_ and NH_3_ production. These results might be due to the antimicrobial properties of carvacrol, its major compound. In contrast, rosemary EO demonstrated a lower effect on CH_4_ mitigation, but it was able to inhibit NH_3_ production. The EOs demonstrated adverse effects on feed degradability and volatile fatty acid production due to their marked and non-specific antimicrobial activity. Cobellis et al. [72] tested various EOs to mitigate methane and ammonia production by rumen microbiota. The authors demonstrated that EOs with different chemical compositions have different effects on the same microbes. The EOs containing phenolic compounds (e.g., carvacrol) or a carbonyl (e.g., cinnamaldehyde) have a stronger effect on microbial populations than EOs that contain monoterpenes. Moreover, interactions among dissimilar EOs and different EO components may influence their activity on microbial populations. Comparing EOs and aqueous extracts (AEs) using the in vitro gas production technique, Molho-Ortiz et al. [131] observed that garlic, cinnamon, and rosemary EOs negatively affected ruminal fermentation. The garlic and cinnamon EOs reduced methane production by 64.7% on average when compared to the other treatments. The AEs of all species showed smaller effects on ruminal fermentation, suggesting that water extraction methods may be inappropriate for obtaining the secondary compounds that are present in different species and that impact rumen fermentation parameters, such as gas and methane production. Zhou et al. [132] studied the effects of different inclusion rates of oregano essential oil (OEO) on ruminal in vitro fermentation parameters, such as total gas and methane production and bacterial communities. The authors considered the following treatments: control—0 mg/L of OEO (CON); 13 mg/L (OEO1); 52 mg/L (OEO2); 91 mg/L (OEO3); and 130 mg/L (OEO4), each incubated with 150 mL of buffered sheep rumen fluid and 1.2 g of substrate for 24 h using the Ankom in vitro gas production system. With an increase in OEO inclusion rates, the digestibility of dry matter and structural carbohydrates increased quadratically. The ammonia-nitrogen concentrations decreased at a quadratic rate with increasing OEO inclusion rates. Total VFA, acetate, propionate, butyrate, valerate, and isovalerate concentrations decreased linearly with increasing OEO inclusion rates. Total gas production levels showed a quadratic response, and methane production showed the lowest level at 91 mg/L (OEO3). As determined by 16S rRNA sequencing, the α-biodiversity of ruminal bacteria was similar among OEO inclusion rates. Increasing OEO inclusion rates linearly increased the relative abundance of *Prevotella* and *Dialister* bacteria. Tawfeeq et al. [133] investigated the effects of organic, watery, and alcoholic extractions of peppermint and rosemary leaves on in vitro ruminant digestibility. Rumen liquor was collected from slaughtered sheep. The addition of 200 mg of organic extractions for peppermint and rosemary significantly decreased dry matter digestibility (DMD) and organic matter digestibility (OMD) compared to the control diet (0 mg) after 48 h of incubations. Roy et al. [134] investigated the modulatory effects of various EOs on rumen fermentation patterns in vitro using a wheat straw-based diet (concentrate: wheat straw ratio of 50:50). The study evaluated four EOs (cinnamon, garlic, oregano, and rosemary) at concentrations of 0, 30, 300, and 600 mg/L of total culture fluid, utilizing the in vitro gas production technique with buffalo rumen liquor. The results showed that all EOs significantly reduced gas production at a concentration of 600 mg/L. Notably, garlic oil also decreased total gas production at a concentration of 300 mg/L. The highest concentration of most oils reduced methane production, with garlic oil at 600 mg/L showing the greatest reduction. Additionally, oregano oil at 600 mg/L significantly decreased ammonia-N concentration. Higher concentrations of EOs generally led to a significant decrease in dry matter degradability. Overall, supplementing a wheat straw-based diet with various EOs modulated rumen fermentation, reduced methane and ammonia-N production, and improved nutrient utilization.

## 7. Economic Implications of Medicinal Plants in Ruminant Feeding

Economic analysis is essential for measuring the profitability of any production system, whose success depends mainly on costs, income, and return on invested capital, and for identifying potential failures. The analysis helps to identify possible obstacles to the activity, such as fluctuations in market prices, and facilitates the allocation of production factors (land, labor, and capital), teaching the producer greater rationality in management and business planning. In ruminant farms, animal feed represents the main production cost; the diets for the dairy production system essentially include feeds combined with a concentrate, the utilization of which depends mainly on the quality of the feed and the genetic potential of the animals [135,136]. In this context, the use of medicinal plants or their extracts in the feeding of ruminants could represent an interesting opportunity to improve the activity of rumen microorganisms, thus improving animal health and productive and qualitative performance. However, the increasing demand for these solutions could represent an ecological risk; indeed, profitable exploitation and commercialization of medicinal plants require a coherent knowledge of their demand and production systems or a value chain analysis in order to avoid overexploitation and consequent environmental degradation. In this regard, Volenzo and Odiyo [137] conceived a framework for a systematic understanding of sustainability in the value chains of medicinal plant resources, promoting the participation of local actors. Indeed, both the marketing and promotion of medicinal plants require significant capital investment, which could lead to a possible dominance of the global value chain by a few countries, whose monopoly negatively affects outcomes and encourages biopiracy, consolidating the risk of rent dispersion. As suggested by Asigbaase et al. [138], the economic value of medicinal plants cannot be ignored as ethnobotanicals are an important source of income and an interesting opportunity for employment for millions of people in both developing and industrialized countries, significantly supporting the gross domestic product [139]. According to Sofowora et al. [140], in five years, the sales of medicinal plants doubled and tripled in China and India, respectively, with a 25% increase in Europe. In addition, Fortune Business Insights [141] estimates a global projection of medicinal plant products at about $400 billion, growing at a rate of 15 to 25% per year [141], while the WHO [142] suggested that by 2050, the trade will be up to about US$ 5 trillion. The exponential increase in global market demand for medicinal plants generates an economic appeal with a significant increase in plant collection, which could compromise their survival [137]. The medicinal plants do not represent an economical additive in ruminant feeding, as it would seem based on their ease and low cost of production. This is due to the complex, time-consuming, and expensive extraction process required to obtain bioactive compounds [143]. In addition, in practice, it may not be economical to feed most herbs directly to animals, especially on a large scale. Therefore, the utilization of herbal residues should be promoted, as well as research on low-cost and simple extraction processes, taking into account the variability in raw plant quality and laboratory methods. In fact, the safe future use of medicinal plants requires highly standardized measures. In the case of oregano, its utilization for cattle feeding has been strongly suggested due to a decrease in methane emissions by 40%, but it does not have a low cost; consequently, researchers have to find an economically realistic use for livestock. According to de Oliveira et al. [144], the addition of up to 0.8% DM of oregano to the diets of dairy cows yielding 12.9 kg/day of milk was demonstrated to be economically efficient. With regard to garlic as a dietary supplement for dairy cows, Kamra et al. [145] obtained an increase in milk yield of 1.2 L per day, although the authors recommend long-term feeding trials with different doses, feeding regimes, and frequencies for its practical application. In addition, they suggested a combination of garlic with other medicinal plants for a more sustainable application to the livestock. Other authors [146,147,148] have reported an improvement in economic efficiency by using rosemary as a feed additive in lamb diets; indeed, the increase in feed costs was largely offset by the increase in total weight gain and improved animal health, both of which increased farm profitability. Finally, it is important to highlight the growing interest in the use of EOs derived from medicinal plants in ruminant feeding; in this regard, Orzuna-Orzuna et al. [149] performed a simulation analysis, obtaining an increase in economic income by 1.44% while also reducing the environmental footprint of beef cattle by 0.83%.

## 8. Conclusions

This study highlights several key findings regarding the effects of garlic, oregano, and rosemary supplementation on ruminant health, performance, and milk production. Key aspects of dairy ruminant husbandry, such as weaning and oxidative stress response, are positively modulated when garlic, oregano, and rosemary supplements are employed. Although garlic and rosemary have been demonstrated to be more consistent in terms of their ability to enhance the quality of milk, the findings concerning oregano have been more variable across studies. These plants indeed exert a range of effects on the animal organism, which depend on the dosage, administration method, and potential synergies with other botanicals. Therefore, limitations in the application of this practice are associated with the presence of different formulations and experimental conditions, which have led to different outcomes for some parameters. Further research is essential to standardize formulations and determine optimal dosages to maximize the observed benefits. To this end, in vitro studies provide insights into the effects on digestibility and fermentation when various plants in different forms are tested. The growing interest in medicinal herb supplementation highlights the potential for enhancing farm profitability, but careful management is essential to avoid overexploitation and ensure sustainability. Further research is needed to make these practices more cost-effective and widely applicable across livestock production systems.

## Figures and Tables

**Table 1:** Main effects of different types of garlic supplementation in ruminants.

Species	Dose	Formulation	Milk Performance	Weaning	Reference
Cows	5 g/head per day	EO	>milk fat >*cis*-9, *trans*-11		[38,46]
	4.35 or 17.39 g/kg	pure	=milk yield and composition		[39]
	10 mg GP/kg BW; 250 mg/kg body weight	powder		<oxidative stress index <incidence of diarrhea and respiratory disease >BW and FI	[61,62]
Goats	30 and 70 g/kg (DM); 2 mL/day/head	pure; oil	<milk fat		[40,41]
	62.5, 125, and 250 mg	aqueous extract		>BW, red blood cells, lymphocytes, and white blood cells	[63]
Sheep	20 g/kg (DM)	powder	>CLA >omega-3		[43]
	1.5 or 3 g	powder		>BW and FI	[64]

EO: essential oil; GP: garlic powder; BW: body weight; FI: feed intake; DM: dry matter; CLA: conjugated linoleic acid.

**Table 2:** Main effects of different types of oregano supplementation in ruminants.

Species	Dose	Formulation	Milk Performance	Weaning	Reference
Cows	18, 36, and 53 or 7, 14, and 21 g of oregano DM/kg; 250 to 750 g/day; 250 to 750 g/day	dried leaves	=milk composition and FA profile >milk fat content		[71,73,75]
	0.056% extract/DM	extract	<milk fat content <fat/protein ratio		[76]
	30 g/d	leaf powder		>BW gain >feed efficiency >triglycerides <duration of diarrhea	[80]
	60 mg/kg body weight/day	extract		Anticipation of the first rumination	[94]
	12.5 mg/kg body weight	EO		<intensity, incidence, and duration of diarrhea	[96]
Goats	30 g/day	ground oregano plants	=milk yield		[77]
Sheep	1 mL/kg of concentrate feed	EO	=milk yield >milk protein =milk fat and lactose content		[78]

DM: dry matter; FA: fatty acid; BW: body weight; EO: essential oil.

**Table 3:** Main effects of different types of rosemary supplementation in ruminants.

Species	Dose	Formulation	Milk Performance	Weaning	Reference
Cows	28.19 kg/d	Extract	>milk protein content		[106]
	1000 mg/day and 2000 mg/day	EO		>feed intake >ADG >IgG concentration	[115]
		active compounds of rosemary		=IgG and IgA	[118]
Goats	10 g/head/day		>milk yield >milk fat yield >UFA >MUFA >CLA <SFA		[100]
	100, 200, and 400 mg per kg of live BW	aqueous extract		=live weight gain and ADG >white cells and globulin	[117]
Sheep	600 and 1200 mg/head/day	extract	>milk yield >milk fat, lactose, casein, and protein content		[103]
	250 mg/day, 500 mg/day, and 750 mg/day	EO		=live weight gain, ADG, and FI >SOD	[116]

EO: essential oil; BW: body weight; ADG: average daily gain; UFA: unsaturated fatty acid; MUFA: monounsaturated fatty acid; CLA: conjugated linoleic acid; SFA: saturated fatty acid; FI: feed intake; SOD: superoxide dismutase.

## Data Availability

The raw data supporting the conclusions of this article will be made available by the authors upon request.
